# Predicting outcomes of smoking cessation interventions in novel scenarios using ontology-informed, interpretable machine learning

**DOI:** 10.12688/wellcomeopenres.20012.2

**Published:** 2025-10-06

**Authors:** Janna Hastings, Martin Glauer, Robert West, Anna Kleinau, James Thomas, Alison J. Wright, Susan Michie

**Affiliations:** 1 Institute for Implementation Science in Health Care, Faculty of Medicine, University of Zurich, Zürich, Switzerland; 2 School of Medicine, University of St Gallen, St. Gallen, Switzerland; 3 Institute for Intelligent Interacting Systems, Otto-von-Guericke University, Magdeburg, Saxony-Anhalt, Germany; 4 Research Department of Behavioural Science and Health, University College London, London, England, UK; 5 Institute for Simulation and Graphics, Otto-von-Guericke University, Madgeburg, Germany; 6 EPPI-Centre, Social Research Institute, University College London, London, England, UK; 7 Institute of Pharmaceutical Science, King's College London, London, England, UK; 8 Centre for Behaviour Change, University College London, London, England, UK

**Keywords:** behaviour change interventions, Artificial Intelligence, machine learning, natural language processing, prediction systems, information extractions, ontologies, evidence synthesis

## Abstract

**Background:**

Systematic reviews of effectiveness estimate the relative average effects of interventions and comparators in a set of existing studies *e.g.,* using rate ratios. However, policymakers, planners and practitioners require predictions about outcomes in novel scenarios where aspects of the interventions, populations or settings may differ. This study aimed to develop and evaluate an ontology-informed, interpretable machine learning algorithm to predict smoking cessation outcomes using detailed information about interventions, their contexts and evaluation study methods. This is the second of two linked papers on the use of machine learning in the Human Behaviour-Change Project.

**Methods:**

The study used a corpus of 405 reports of randomised trials of smoking cessation interventions from the Cochrane Library database. These were annotated using the Behaviour Change Intervention Ontology to classify, for each of 971 study arms, 82 features representing details of intervention content and delivery, population, setting, outcome, and study methodology. The annotated data was used to train a novel machine learning algorithm based on a set of interpretable rules organised according to the ontology. The algorithm was evaluated for predictive accuracy by performance in five-fold 80:20 cross-validation, and compared with other approaches.

**Results:**

The machine learning algorithm produced a mean absolute error in prediction percentage cessation rates of 9.15% in cross-validation, which was lower than the mean absolute error of other approaches including an uninterpretable ‘black-box’ deep neural network (9.42%), a linear regression model (10.55%) and a decision tree-based approach (9.53%). The rules generated by the algorithm were synthesised into a consensus rule set to create a publicly available predictive tool to provide outcome predictions and explanations in the form of rules expressed in terms of predictive features and their combinations.

**Conclusions:**

An ontologically-informed, interpretable machine learning algorithm, using information about intervention scenarios from reports of smoking cessation trials, can predict outcomes in new smoking cessation intervention scenarios with moderate accuracy.

## Introduction

Changing human behaviour at scale is necessary to address many of the challenges facing humankind ^1^. Behavioural science aims to discover better ways of achieving this. Much of this research involves evaluating behaviour change interventions in randomised controlled trials whose findings need to be synthesised and interpreted (see Supplementary file 1: Table S1 ^2^ for a glossary of terms and abbreviations). Meta-analyses and meta-regressions evaluating intervention effectiveness usually estimate the average effects of intervention packages reviewed compared with comparators which may be other interventions such as ‘usual care’ or ‘follow-up only’. A small number of studies have used machine learning to attempt to predict individual outcomes in trials ^3– 5^. However, what policymakers, planners and practitioners require are predictions about the outcomes of interventions in scenarios that contain specific combinations of features that have not been directly evaluated. This is the second of two linked papers. In the first paper, we described an initial unsuccessful attempt to develop a machine learning model for prediction smoking cessation outcomes ^6^. This paper describes a new methodology for predicting the outcomes of potentially novel combinations of features of intervention scenarios using information about intervention content and delivery, populations, and settings.

Current approaches to using evidence synthesis and meta-analysis to predict outcomes do not take account of the high level of context dependency in behaviour. The same intervention package may have different effects in different populations or settings ^7^. In addition, it is rare to be able to disaggregate intervention components at a granular level to assess the extent to which particular components contribute at all, or operate additively, synergistically or in competition with each other ^8, 9^. Moreover, studies are almost never completely replicated and differences in methods can have a major impact on the findings ^10^. The result is that many systematic reviews and meta-analyses are forced to make overly general conclusions or are unable to draw conclusions because of heterogeneity of outcomes ^11^.

A solution is to link features of interventions, populations, settings and methodology in study arms of randomised trials with outcomes ( *e.g.,* percentage of smokers who stop smoking for at least six months) and use machine learning (ML) to learn associations between the features and outcomes. Randomised trials provide a useful starting point for this kind of analysis because they tend to have more rigorous and detailed information than is found in observational studies and the quality control of data collection can be expected to be higher because of guidelines on the conduct of trials ^12^. A limitation is that the study samples and methods may reduce generalisability.

An initial attempt to use ML to predict outcomes in smoking cessation trials applied a deep-learning algorithm and was not successful ^6^. It did not take advantage of the ontological structure of the data, used a ML approach that may not have been optimal for the data set, and produced a ‘black box’ solution that was not interpretable.

We aimed to develop a new approach to the prediction of smoking cessation outcomes based on interpretable ML from the trial research findings, which was ontologically-informed, that is, constrained by semantic relationships within the Behaviour Change Intervention Ontology (BCIO) ^13^. This paper describes the development and evaluation of this ontologically-informed interpretable ML prediction system and its application to the task of predicting smoking cessation outcomes across a wide range of scenarios.

## Methods

### Dataset annotation

We started with 512 published reports of randomised controlled trials (RCTs) of smoking cessation interventions identified from the Cochrane database of systematic reviews and described in detail elsewhere ^14, 15^. The main resource for establishing this dataset of published reports were systematic reviews from the Cochrane Library ^16^. The library was searched for Cochrane systematic reviews on smoking cessation and all reviews were considered for inclusion in the corpus. Systematic reviews report the entire list of included studies in the review and also a list of relevant but not included studies. Studies from which outcome data can reliably be extracted are included in a meta-analysis. From every systematic review we selected only those studies that were included in the meta-analysis. This was done to allow us to eventually compare the results of any automated meta-analysis system to the ground truth results, which were produced from the review.

A second source of papers, IC-SMOKE, is a systematic review project of behavioural smoking cessation trials, which is funded by Cancer Research UK ^9^.

We used the following criteria for the selection of the papers:

- They are randomised control trials (RCT);
- They are included studies in a systematic review on smoking cessation;
- They are included in a meta-analysis in a systematic review on smoking cessation;
- They have a behavioural outcome value at a pre-defined follow up time point (in the case of smoking cessation, the percentage of participants who stopped smoking)

Entities specified within the BCIO ^13^ were identified from these reports manually using the web-based EPPI-Reviewer tool version 4 ^17^, a software program for managing and analysing data in all types of systematic review. An open alternative to this software used for annotation is PDFAnno ^18^. Papers were annotated manually using a coding scheme based on the ontology to tag pieces of text in PDF documents with codes relating to entities in the ontology and link these to a study arm (group receiving a particular intervention or a comparator). For example, the phrase “44.5 years” might be annotated as characterising the “average age of participants” in a study arm, where average age corresponds to an entity in the BCIO. As well as capturing the value of the entity, annotators also recorded the surrounding text (context) ( *e.g.,* the sentence around “44.5 years”).

Entities were divided into three types depending on how they were annotated:

1.   Presence-absence type: the presence of a particular entity in an intervention ( *e.g.,* ‘goal-setting behaviour change technique’).

2.   Value type: a single numerical value of a variable ( *e.g.,* 26.7 for the variable ‘percentage of participants achieving 12-months of smoking abstinence’).

3.   Complex type: two or more numerical values of entities linked under a parent class ( *e.g.,* 34.4 for the entity ‘percentage of White participants’, 4.5 for the entity ‘percentage of Asian participants’ under the parent class of ‘ethnic group’).

Initially, two annotators independently extracted entities from the papers and discussed their annotations to resolve any discrepancies. Once acceptable inter-rater reliability had been established using the coding scheme, every fifth paper was double-coded to check for annotator ‘drift’.

### Dataset pre-treatment, cleaning and validation

The initial annotations extracted from EPPI-Reviewer contained a set of 123 features and 1098 intervention arms in 512 studies.

Additional features were added to split features with multiple categorical values into separate columns, and in order to separate numeric features into distinct columns for different value ranges. After this process, the dataset had 153 features across the 1098 intervention arms. Pregnancy trials and relapse prevention trials were removed, leaving 1039 intervention arms. Trials that re-used data from other studies or which did not report outcome values were also removed, leaving 971 intervention arms across 405 studies. Finally, features which were used fewer than 30 times or were inconsistently described were removed from the dataset, while for intervention content features aggregate features were added to represent hierarchical groupings.

Subsequent to data annotation, the dataset was subjected to data cleaning and transformation to produce a dataset suitable for use in the prediction task. The full sequence of data cleaning and treatment steps are described in Supplementary file 1: Appendix A ^2^. In brief, initial pre-treatment removed artifacts from the PDF processing, converted numeric values that had been specified as text to standard numeric representation, standardised rounding, and encoded presence or absence as 1 and 0 respectively.

Annotations that contained different values within the same annotation were separated into different distinct features. For example, health professionals were sometimes further specified *e.g.,* as doctor or nurse. The description of control conditions was heterogeneous and was harmonised to a single feature ‘control’.

A precondition for the rules-based approach we developed ^19^ is that all input data features are converted into binary form (‘binarised’). Thus, categorical variables in the dataset were recoded into separate columns per value, and continuous (quantitative) variables were binarised by selecting ranges using one of several different approaches depending on the meanings of the values:

- Separation into meaningful semantic categories, *e.g.,* for our case study we transform mean age values into child, young adult, older adult, and elderly, delineated with a ‘fuzzy’ (see glossary for meaning) membership operator since the boundaries between categories are not rigid.
- Fixed-width categories, delineated with a ‘fuzzy’ membership operator. For example, we divided the number of times tobacco smoked per day into groups of 5 corresponding to <5, <10, <15, ... <50. Note that this formulation creates an ordering because if the value is *e.g.,* 6, then all of <10, <15, ..., <50 will be set on, and if the value is 46, only <50 will be set on.
- Categories selected based on quantiles in the dataset ( *i.e.,* a fixed proportion of the available data in each grouping, rather than fixed range of values), again using the <x formulation to maintain ordering.
- Fixed numeric values, for flagging exact values with a specific meaning, for example 100% female within the population percentage female continuous variable.
- Data-driven clustering was used to determine clusters of data associated with specific value ranges.

As a result of this pre-treatment, additional data columns were added to the overall dataset. Some entities were discarded subsequent to annotation as they were too inconsistently described to be usable ( Supplementary file 1: Table S2, Part A ^2^), or appeared too few times in the dataset: features that had fewer than 30 annotations in the dataset as a whole were removed ( Supplementary file 1: Table S2, Part B ^2^). However, some intervention content attributes were combined into aggregate features using the hierarchy of the Behaviour Change Technique (BCT) Ontology so as not to completely lose the information about the intervention. The aggregation was based on an initial draft of the BCT Ontology. The original features with low numbers of appearances were removed from the dataset while the aggregate features were retained.

Several study reports were removed as (a) they re-analysed data present another study reported in the corpus, or (b) participants were not fully randomised to all arms, or (c) outcome values were not fully specified due, for example, to being shown only in an unannotated figure ( Supplementary file 1: Table S3, Part A ^2^). Pregnancy trials and relapse prevention trials were also removed: the former because there were too few of these and they were too different a population from the remainder of the studies, and the latter because participants were abstinent smokers at baseline rather than smokers so that the outcomes would have a very different meaning ( Supplementary file 1: Table S3, Part B ^2^). 

The ontology contains a class hierarchy, which was used to pre-complete the input data table as follows: if a lower-level feature is ‘on’ ( *i.e.,* set to 1), we ensured that the higher-level feature that subsumed it was also set to ‘on’ in the input dataset. The source code for cleaning and transforming the dataset from the exported EPPI-Reviewer JSON data to the final table used for training the model is available from ^20^.

To assess how far the final data set would provide a potentially useful basis for outcome prediction we compared the mean outcome values for the dichotomised features, looking for reliable differences between the values of variables that would be expected to differ from past research: for example presence versus absence of face-to-face support.

### Outcome prediction

The resulting cleaned dataset was used to train a model for prediction of outcomes of novel intervention scenarios. Outcomes were percentages, representing the reported percentage of participants in a study arm who fulfilled the abstinence criteria.

Outcome prediction is a new task in meta-analyses and meta-regressions in behavioural science, with evidence synthesis primarily using forms of statistical regression to estimate differences in outcomes in existing data sets attributable to interventions or population or setting features. These are expressed as regression weights, odds ratios or similar parameters and they are derived from the data set used. Our task was to predict actual outcomes ( *e.g.,* % achieving smoking abstinence) in an unseen test dataset, using a model trained with data in a training set. This is a much more challenging task because it requires generalising models to novel scenarios by building a model using all available data, including possibly complex causal interactions between predictors.

### Algorithm development

In predicting outcome values for intervention scenarios, explanations of the predictions are as important as the accuracy of the system, since the intended users are practitioners, planners and policy-makers who will use the information gained from the evidence in order to make recommendations and therefore require transparency and accountability ^21^. Deep neural networks typically operate as ‘black boxes’ without giving explanations as to why specific predictions have been made. Explainability approaches do exist that are able to connect the predictions of black box models to feature importances, however, these approaches may give variable results lacking consistency and reproducibility ^22^. Thus, there is a need for ‘glass-box’ explainable ML frameworks for making predictions and recommendations that can transparently provide explanations in a form that matches the way users construe the scenarios ^19, 23, 24^.

Current ML methods for predicting intervention outcomes each have different limitations ^21^. Straightforward application of symbolic ontology-based approaches to learning from data is not well suited for the quantitative task of predicting intervention outcomes. Traditional symbolic learning approaches such as rules or decision trees lead to explanations that can be overly complex and cannot readily be ranked by their quantitative impact on the outcome variable. A deep neural network approach can provide good predictions but cannot be interpreted. Thus, we aimed to develop a ‘best of both worlds’ hybrid predictive approach that combined aspects of the symbolic and neural approaches. Rules-based systems are inherently explainable because the features appear transparently in the rules. Our approach builds on systems that are able to learn rules from data ^19^.

The basic idea of our approach was to learn a maximally predictive and explanatory set of rules through an optimisation process that hides parts of the combinations of features that are not relevant to a particular rule. Our system starts from a set of rules, each of which includes all features in the dataset and their absence. For example, the feature “not nurse” is set to “on” if it is known that no nurse was involved in delivering the intervention. A weight is then attached to each feature in each rule as well as the overall rule. Features with small weights will not influence the rule as much as ones with larger weights. A fit is computed for a specific feature set, from the combination of feature weights and the overall rule weight. The fits of all the rules in the rule set are then combined to form the final prediction. The weights are initially random, and are iteratively updated and tuned as a part of the training process during which features whose weights are below a predetermined threshold are dropped to result in a smaller set of shorter rules. For a more detailed technical description of this approach, see our technical report ^19^.

### Semantic penalties

The basic rule induction system as described thus far was able to successfully derive rules from data and make predictions. However, while the rules it generated were transparent, they were not always readily interpretable, as the weights attached to features could take arbitrary values, and the rules could contain a large number of features. Thus, in order to force the system to learn shorter rules with a smaller number of features, additional penalties were introduced into the training process for (i) the lengths of rules in terms of number of features, and (ii) the ‘crispness’ of the features within rules (how close the weight was to 1.0),

These penalties improved the interpretability of the learned rules significantly. However, the resulting rule set still included rules that did not make sufficient sense according to a review by domain experts. Thus, in addition to the penalties introduced for the length of the rule and crispness of features, the classification and relationships from the ontology were harnessed to introduce ontology-based, semantic constraints. These took two forms:

1. Relationships between entities in the ontology were used to reduce rule length and enhance rule interpretability. Intuitively, a rule that has two features, such as ‘somatic delivery’ and pharmacological support, which are related in such a way that the presence of one feature implies the other must be present as well, can be reduced to a rule with one feature, *i.e.,* pharmacological support. This is because of the relationship in the ontology that every intervention with pharmacological support is also one with a somatic delivery mode. Using these relationships from the ontology resulted in shorter, more readable rules.
2. The ontology also contains axioms regarding negative dependencies between features. For example, an intervention that has the feature ‘buproprion’ (a pharmacotherapy that is administered in the form of a pill) cannot have the feature ‘not pill’. These dependencies can, for instance, be expressed as ‘disjointness’ axioms. A constraint was thus added to force rules not to include features that contradict each other semantically based on the ontology.

### Training procedure

In order to train a set of rules from the available data, a set of rules is first created starting with random weights, and then the correct weights are trained iteratively using optimisation with backpropagation.

The model was initialised with a set of 200 rules. Singleton rules for each non-negated ( *i.e.,* non-absent) feature were initialised based on the weights from a simple linear regression, and the remaining ones were initialised randomly.

### Algorithm evaluation

The accuracy of the prediction algorithm was evaluated using five-fold cross-validation. That is, we implemented five iterations, in each of which we selected 80% of 405 studies to use as a training set, which was itself split into a training dataset and a validation dataset, and then attempted to predict the outcomes for the remaining 20%. The dataset was split into five parts, such that after five iterations of the cross-validation, all outcome values had been used once in a testing set in a separate run of the cross-validation. In each iteration, the model was trained for at least 400 epochs (training runs), after which the training was stopped as soon as the loss (including penalties) did not further improve on the validation set. The final evaluation for each of the five validation iterations was then conducted on the 20% hold-out test set that had not been seen before in that validation iteration, and this procedure was repeated five times. 

The accuracy metric for the prediction algorithm was the mean absolute error, where the error of prediction is the difference between the predicted and annotated outcome value. This is a standard metric for evaluating predictions of this kind ^25^.

We evaluated our algorithm by comparison with four different algorithms from different families of ML approaches:

- Mixed-effects linear regression: As baseline we used a mixed-effect linear regression model with a random effect for study and fixed effects for all other variables.
- Grand mean: We compared the predictive performance to a prediction that always only predicts the mean of the training dataset.
- Random forest: As a comparator for an alternative interpretable approach, we used a random forest of 50 trees with a maximal depth of 3 layers and 5 leaf nodes.
- Deep neural network: As a comparator for the state-of-the-art best ‘black box’ quantitative predictive model, we used a feed-forward neural network with 77 input neurons and 3 hidden layers with 154, 77 and 38 neurons, trained for 100 epochs using the Adam optimizer. We did not attempt any interpretability approaches on this model.

In order to compare the performance of the models we executed a non-parametric Mann-Whitney U test of the absolute errors to compare the distributions of the errors between the rule-based model and the other models. We estimated the Bayesian likelihood of superior accuracy of the interpretable machine learning prediction algorithm compared with the other approaches assuming uninformative priors using one-tailed p value from the Mann-Whitney test.

### Consensus model

The full dataset was used to train a deployment-ready version of the model. However, as the learned rules were under-constrained by the available data given the ratio of data points to parameters, it was possible to stochastically generate multiple different sets of rules which were equally predictive. Therefore, we aimed to develop an approach to generate a final deployment set of consensus rules from multiple training runs. We executed the rule induction 1,000 separate times and compared the resulting generated rules. We assumed that the feature combinations in rules were drawn randomly from a hypergeometric distribution which estimates the probability to draw a specific set of k items when drawing m items from a collection containing n elements. We assume a distribution in which k is the length of a specific rule, m is the mean rule length over all models and n is the number of features. Then we selected all feature combinations that were present in more than 130% of the expected number of appearances in the 1,000 training runs.

After creating the combined consensus rule set, the weights of the final set of rules were fine-tuned in a final training run to adjust the rule set weights to an overall predictive set using all the training data.

As all the data were used to develop the final consensus model, we could not evaluate the consensus model using cross-validation. Instead, we qualitatively evaluated the model by exploring how the predictions it made differed in ways that would be expected from prior knowledge. Thus, from prior knowledge it was expected that:

- Older smokers would be predicted to have higher success rates overall than younger smokers ^26^.
- Smokers with higher daily cigarette consumption would be predicted to have lower success rates than those with lower cigarette consumption ^26^.
- Predicted success rates that were biochemically verified would be lower than those that were unverified ^27^.
- Predicted success rates based on point-prevalence abstinence would be higher than those based on continuous abstinence ^28^.
- For a population with mean age of 50 years, 50 percent women, smoking an average of 10 cigarettes per day with follow-up at 26 weeks, biochemical verification and assessing continuous abstinence:
  ○ Predicted success rates would be higher in smokers receiving face-to-face delivery by a health professional of a combination of behavioural support BCTs that include: problem solving, reducing negative emotions, self-monitoring of behaviour, behavioural substitution and reducing prompts and cues ^29^.
  ○ Over and above this, predicted success rates in smokers given NRT, bupropion, or varenicline would be higher than those given placebo or no pharmacotherapy ^30^.

The question arises as to how consistent the agreement between different consensus models is. For this purpose, we consider two different types of agreement: First, the left-hand sides of the rules can be compared. To quantify this, we consider the left-hand sides of the learned rules as sets of the individual conjuncts. A model { *nurse*∧ *patch*→0.8, *nurse*→1.5} would accordingly be regarded as a set {{nurse, patch}, {nurse}}. The Jaccard index can thus be used as a measure of the similarity between two models. In order to gain more precise insights into the consensus achieved, we trained a total of 30 batches of 1000 models each. Each batch was grouped into one consensus model. For this first type of agreement, we have now determined the pairwise similarity of the 30 consensus models.

However, this metric of similarity ignores the right-hand sides of the rules. Two hypothetical models { *nurse*∧ *patch*→0.8} and { *nurse*∧ *patch* → –0.8} would yield a high similarity here, although they would produce very different results. Therefore, we also looked at the distribution of the right-hand sides. For this purpose, for each conjunction C we considered all consensus models *M _i_* that contain a rule { *C* –> *w _i_*} and collected all right-hand sides *W _C_* = { *w _i_* | ( *C* –> *w _i_*) *in* *M _i_*} and determined the standard deviation and mean value for each of these sets.

### Interface development

The resulting consensus model was adopted for deployment in a publicly available user interface.

The prediction interface was developed using a Flask server in Python and a Vue web-based user interface in JavaScript. The interface gives the option to specify baseline features, such as mean population age, and intervention features such as the behaviour change techniques applied. The system then uses the trained model to make two predictions – a control prediction, without intervention content, and an intervention prediction with intervention features. The predicted values are displayed together with the rules that are used in the prediction. The predictions and rules are displayed alongside a graphical representation of the impact of each rule visualised in descending order of absolute impact and separated between positive impact (green bars) and negative impact (red bars), and between control and intervention. The design of the interface was discussed in and informed by feedback from interdisciplinary meetings of the Human Behaviour-Change Project team and by guidance from domain experts on smoking cessation.

## Results

After data cleaning and preparation, the dataset contained 106 features, of which 82 were semantically distinct ( *i.e.,* not binarised to capture a value range of another feature). The 82 semantically distinct features that were used for training the semantic constraints, are shown in Table 1 to Table 5. In some cases, features were grouped under ‘parent features’.

**Table 1.** Intervention features (N=50) used in prediction model.

Feature name ^1^	Definition ^2^	Parent feature (where applicable)
**BCT cluster features**		
Awareness of others BCT	A behaviour change technique that increases awareness of what other people think, do, or feel.	
Personal resources BCT	A behaviour change technique that increases available personal resources.	
Monitoring BCT	A behaviour change technique that involves gathering or using information about performance.	
Consequences BCT	A behaviour change technique that draws attention to or alters the consequences of the behaviour.	
Goal directed BCT	A behaviour change technique that sets or changes goals.	
Alter external stimulus BCT	A behaviour change technique that involves creating, strengthening or reducing an association between the behaviour and an environmental trigger.	
Habit BCT	A behaviour change technique that aims to alter habits.	
Mental representation BCT	A behaviour change technique that aims to alter mental representations.	
Social support BCT	A behaviour change technique that involves taking steps to secure or deliver the support or aid of another person.	
Restructure the environment BCT	A behaviour change technique that alters the environment in which the behaviour is, or would have been, performed in a way that facilitates or impedes the behaviour.	
**Individual BCT features**		
1.1. Goal setting (behaviour)	Set or agree on a goal defined in terms of the behaviour to be achieved.	Goal directed BCT
1.2 Problem solving	Analyse, or prompt the person to analyse, factors influencing the behaviour and generate or select strategies that include overcoming barriers or increasing facilitators.	Goal directed BCT
1.4 Action planning	Prompt detailed planning of performance of the behaviour.	Goal directed BCT
1.5 Review behaviour goal(s)	Review behaviour goal(s) jointly with the person and consider modifying goal(s) or behaviour change strategy in light of achievement.	Goal directed BCT
1.8 Behavioural contract	Create a written specification of the behaviour to be performed, agreed on by the person, and witnessed by another.	Goal directed BCT
1.9 Commitment	Ask the person to affirm or reaffirm statements indicating commitment to change the behaviour.	Goal directed BCT
2.1 Monitoring of behaviour by others without feedback	Observe or record behaviour with the person’s knowledge as part of a behaviour change strategy.	Monitoring BCT
2.2 Feedback on behaviour	Monitor and provide informative or evaluative feedback on performance of the behaviour *.*	Monitoring BCT
2.3 Self-monitoring of behaviour	Establish a method for the person to monitor and record their behaviour(s) as part of a behaviour change strategy.	Monitoring BCT
2.7 Feedback on outcome(s) of behaviour	Monitor and provide feedback on the outcome of performance of the behaviour.	Monitoring BCT
3.1 Social support (unspecified)	Advise on, arrange or provide social support or non-contingent praise or reward for performance of the behaviour *.*	Social support BCT
3.2 Social support (practical)	Advise on, arrange, or provide practical help for performance of the behaviour.	Social support BCT
4.1 Instruction on how to perform the behaviour	Advise or agree on how to perform the behaviour.	Mental representation BCT
4.2 Information about Antecedents	Provide information about antecedents ( *e.g. social and environmental* * situations and events, emotions, cognitions)* that reliably predict performance of the behaviour.	Mental representation BCT
4.5. Advise to change behaviour	Advise, encourage or tell the person to change the behaviour.	Awareness of others BCT
5.1 Information about health consequences	Provide information (e.g. written, verbal, visual) about health consequences of performing the behaviour	Consequences BCT
5.2 Salience of consequences	Use methods specifically designed to emphasise the consequences of performing the behaviour with the aim of making them more memorable (goes beyond informing about consequences).	Consequences BCT
5.3 Information about social and environmental consequences	Provide information (e.g. written, verbal, visual) about social and environmental consequences of performing the behaviour.	Consequences BCT
6.1 Demonstration of behaviour	Provide an observable sample of the performance of the behaviour, directly in person or indirectly e.g. via film, pictures, for the person to aspire to or imitate.	Mental representation BCT
6.2 Social comparison	Draw attention to others’ performance to allow comparison with the person’s own performance.	Awareness of others BCT
7.3 Reduce prompts/cues	Withdraw gradually prompts to perform the behaviour	Alter external stimulus BCT
8.1 Behavioural practice/ rehearsal	Prompt practice or rehearsal of the performance of the behaviour one or more times in a context or at a time when the performance may not be necessary, in order to increase habit and skill	Habit BCT
8.2 Behaviour substitution	Prompt substitution of the unwanted behaviour with a wanted or neutral behaviour	Habit BCT
8.7 Graded tasks	Set easy-to-perform tasks, making them increasingly difficult, but achievable, until behaviour is performed	Habit BCT
9.1 Credible source	Present verbal or visual communication from a credible source in favour of or against the behaviour.	Awareness of other people’s thoughts, feelings and actions BCT
9.2 Pros and cons	Advise the person to identify and compare reasons for wanting (pros) and not wanting to (cons) change the behaviour.	Consequences BCT
10.1 Material incentive (behaviour)	Inform that money, vouchers or other valued objects will be delivered if and only if there has been effort and/or progress in performing the behaviour.	Consequences BCT
10.4 Social reward	Arrange verbal or non-verbal reward if and only if there has been effort and/ or progress in performing the behaviour.	Consequences BCT
11.1 Pharmacological support	Provide, or encourage the use of or adherence to, drugs to facilitate behaviour change.	
11.2 Reduce negative emotions	Advise on ways of reducing negative emotions to facilitate performance of the behaviour.	Personal resources BCT
12.3 Avoidance/reducing exposure to cues for the behaviour	Advise on how to avoid exposure to specific social and contextual/physical cues for the behaviour, including changing daily or weekly routines.	Restructure the environment BCT
12.5 Adding objects to the environment	Add objects to the environment in order to facilitate performance of the behaviour.	Restructure the environment BCT
12.6 Body changes	Alter body structure, functioning or support directly to facilitate behaviour change	Personal resources BCT
13.2 Framing/reframing	Suggest the deliberate adoption of a perspective or new perspective on behaviour (e.g. its purpose) in order to change cognitions or emotions about performing the behaviour.	Mental representation BCT
**Pharmacological support** ** features**		
NRT	Nicotine Replacement Therapy (nicotine transdermal patch, nicotine chewing gum, nicotine lozenge, nicotine inhalator, e-cigarette)	11.1 Pharmacological support
Bupropion	An atypical antidepressant found to aid smoking cessation, taken as a tablet.	11.1 Pharmacological support
Varenicline	A nicotine receptor partial agonist found to aid smoking cessation, taken as a tablet.	11.1 Pharmacological support
Placebo	A tablet or device with no-pharmacologically active ingredient.	11.1 Pharmacological support
**Intervention package features**		
Brief advice	An intervention package consisting of advice to stop smoking and advice on how to succeed delivered in a single session of less than 30 minutes.	
Motivational Interviewing	A directive, client-centred counselling intervention package for eliciting behaviour change by helping clients to explore and resolve ambivalence.	

^1^These labels and groupings are from an early version of the Behaviour Change Technique Ontology that is close to the original BCTv1 taxonomy from which it was developed. ^2^For ease of reading these are informal definitions rather than the strict ontological definitions.

**Table 2.** Mode of Delivery features (N=12) used to train prediction model.

Feature name ^1^	Definition ^2^	Parent feature (where applicable)
Digital content type	Any form of intervention delivery using digital media, including videos.	
Website / Computer Program / App	Forms of digital media involving computer programs.	Digital content type
Digital content type	Any form of intervention delivery using digital media, including videos.	
Website / Computer Program / App	Forms of digital media involving computer programs.	Digital content type
Text messaging	SMS text messaging support.	
Phone	Support from a human practitioner over the telephone.	
Face to face mode of delivery	Human interactional mode of delivery that involves an intervention source and recipient being together in the same location and communicating directly.	
Group-based mode of delivery	Mode of delivery that involves three or more people in the location where the intervention is delivered.	
Printed material mode of delivery	Informational mode of delivery that involves use of printed material. (e.g. Leaflets, booklets and books.)	
Somatic mode of delivery	Mode of delivery that involves devices or substances that alter bodily processes or structure.	
Pill mode of delivery	Alimentary mode of delivery that involves swallowing of a pill or oral capsule. (Only applies to bupropion, varenicline and placebo.)	Somatic mode of delivery
Patch	Ingestion mode of delivery that involves ingestion of a chemical through the skin. (Only applies to NRT)	Somatic mode of delivery
Lozenge	Ingestion mode of delivery that involves absorption of a chemical through the lining of the buccal cavity. (Only applies to NRT)	Somatic mode of delivery
Gum	Ingestion mode of delivery that involves chewing of a soft material. (Only applies to NRT)	Somatic mode of delivery

^1^These labels and groupings are from an early version of the Behaviour Change Intervention Ontology. ^2^For ease of reading these are informal definitions rather than the strict ontological definitions.

**Table 3.** Source of delivery features (N=4) used in the prediction model.

Feature name ^1^	Definition ^2^	Parent feature (where applicable)
Health Professional	A practitioner in a healthcare role.	
Psychologist	A practitioner with a degree-level or higher qualification in psychology.	Health Professional
Doctor	A practitioner with a qualification as a doctor.	Health Professional
Nurse	A practitioner with a qualification as a nurse.	Health Professional

^1^These labels and groupings are from an early version of the Behaviour Change Intervention Ontology. ^2^For ease of reading these are informal definitions rather than the strict ontological definitions.

**Table 4.** Setting feature (N=1) used in the prediction model and population features (N=6) used in the prediction model.

Feature name	Definition ^1^
Healthcare facility	A facility that is administered by a health care organisation for the purpose of providing health care to a patient population ( *e.g.,* a hospital or clinic.)
Mean age	The mean age for the study participants in a study arm.
Mean number of times tobacco used (Quantity)	The mean number of cigarettes smoked daily prior to starting the intervention.
Mean number of times tobacco used (Reported)	Whether the mean number of times tobacco was used was reported in the study.
Patient role	The smoker being a hospital or general practice patient (either inpatient or outpatient).
Proportion identifying as female gender	Percentage of the study population who reported identifying as female gender
Proportion identifying as male gender	Percentage of the study population who reported identifying as male gender

^1^For ease of reading these are informal definitions rather than the strict ontological definitions.

**Table 5.** Outcome features (N=9) used in the prediction model.

Feature name	Definition ^1^
Control	The designated control condition of a study.
Abstinence: Continuous	Abstinence was assessed from the target quit date or soon afterwards up to the follow-up point.
Abstinence: Point Prevalence	Abstinence was assessed for the past week at the follow-up point.
Biochemical verification	Test for nicotine, a nicotine metabolite, thiocyanate in urine, blood or saliva, or carbon monoxide in exhaled air to confirm abstinence at the follow-up point.
Whether follow-up duration reported	Check on whether follow-up duration is reported for the outcome value.
Follow-up duration	Duration of follow up in weeks
Sample size	The reported number of participants in a study arm/analysed as receiving the intervention.
Pharmaceutical company competing interest	Whether there is a declaration of author support from or involvement of a pharmaceutical company.
Pharmaceutical company funding	Whether there is a declaration of study funding from a pharmaceutical company.

^1^For ease of reading these are informal definitions rather than the strict ontological definitions.

The final dataset used for model training is available from ^20^. 

Comparison of the mean values of the dichotomised variables in the final dataset showed reliable differences in outcomes for features that would be expected from prior research to show differences ( see Supplementary file 2 for a full table of means) ^2^. For example, presence of the ‘Problem Solving BCT’ was associated with a 4.2 percentage point increase in outcome on average. Presence of ‘Self-monitoring of behaviour BCT’ was associated with a 5.4 percentage point increase in outcome. Presence of the ‘Varenicline pharmacological support feature’ was associated with an 8.0 percentage point increase in outcome. Presence of the ‘Face-to-face support feature was associated with a 5.7 percentage point increase in outcome. Therefore, the dataset appeared to show enough patterning in the data potentially to provide a basis for the ML prediction process.

## Algorithm evaluation

**Figure 1.**
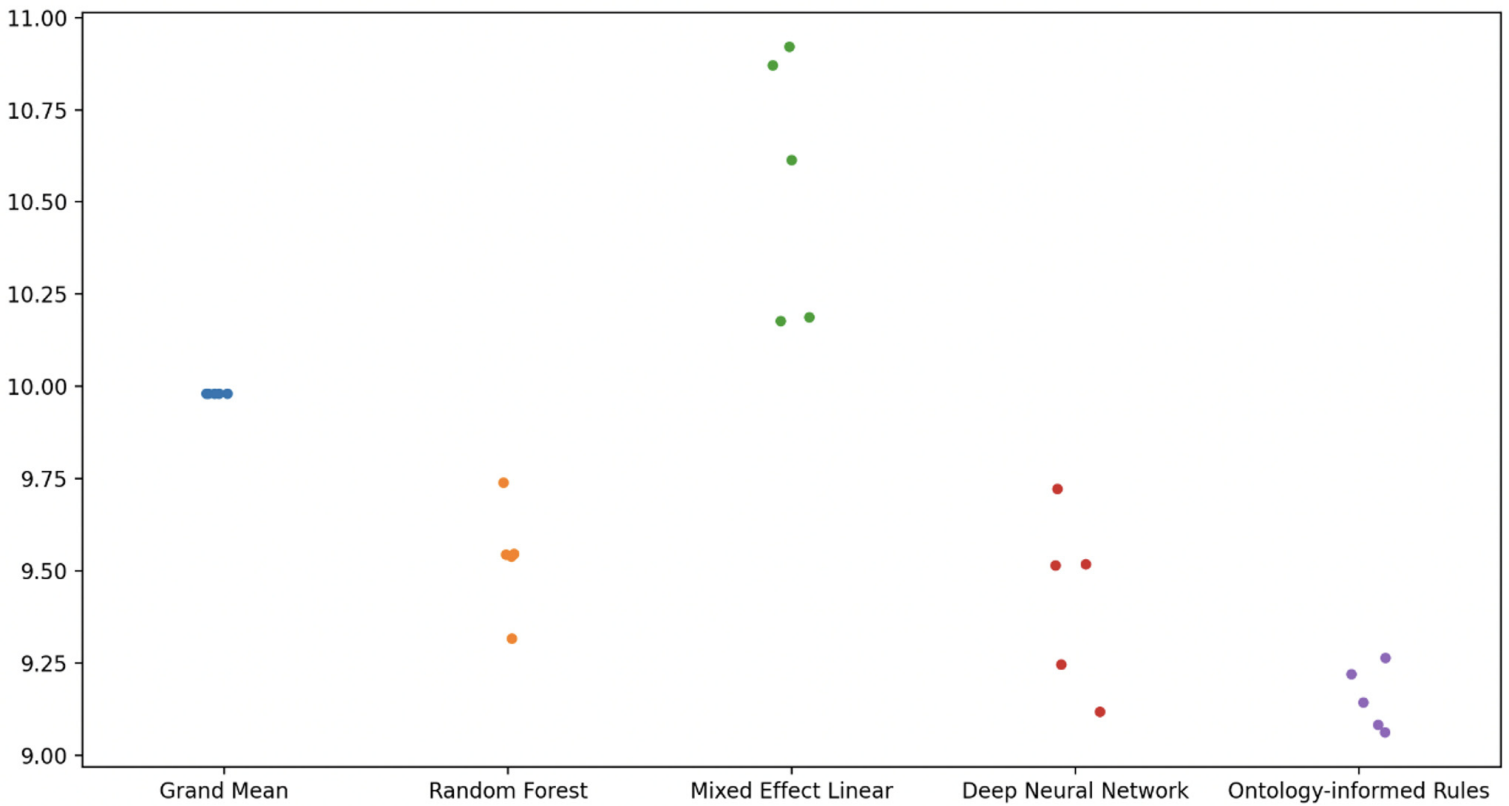
Mean Absolute Errors from cross-validation of five separate runs.

To evaluate the novel algorithm for prediction, we compared the predictive performance in five-fold cross-validation with other algorithms. The mean of the mean absolute errors for the five-fold cross-validations are shown in Table 6 and Figure 1.

**Table 6.** Mean absolute errors from cross-validation for four machine learning approaches and a prediction based on the grand mean of the training dataset.

Algorithm	Mean Absolute Error	Bayesian likelihood of superior accuracy
Grand mean	9.98	
Random forest (ensemble of decision trees)	9.53	0.92
Mixed-effect linear regression	10.55	0.99
Deep learning neural network	9.42	0.96
Ontology-informed rules (our novel approach)	**9.15**	

In terms of the mean absolute errors, our novel algorithm out-performed other approaches in cross-validation, including a deep learning neural network, and a statistical comparison indicated at least a 92% likelihood for the hypothesis that the interpretable machine learning approach is generally superior to the other approaches.

### Consensus model validation

**Figure 2.**
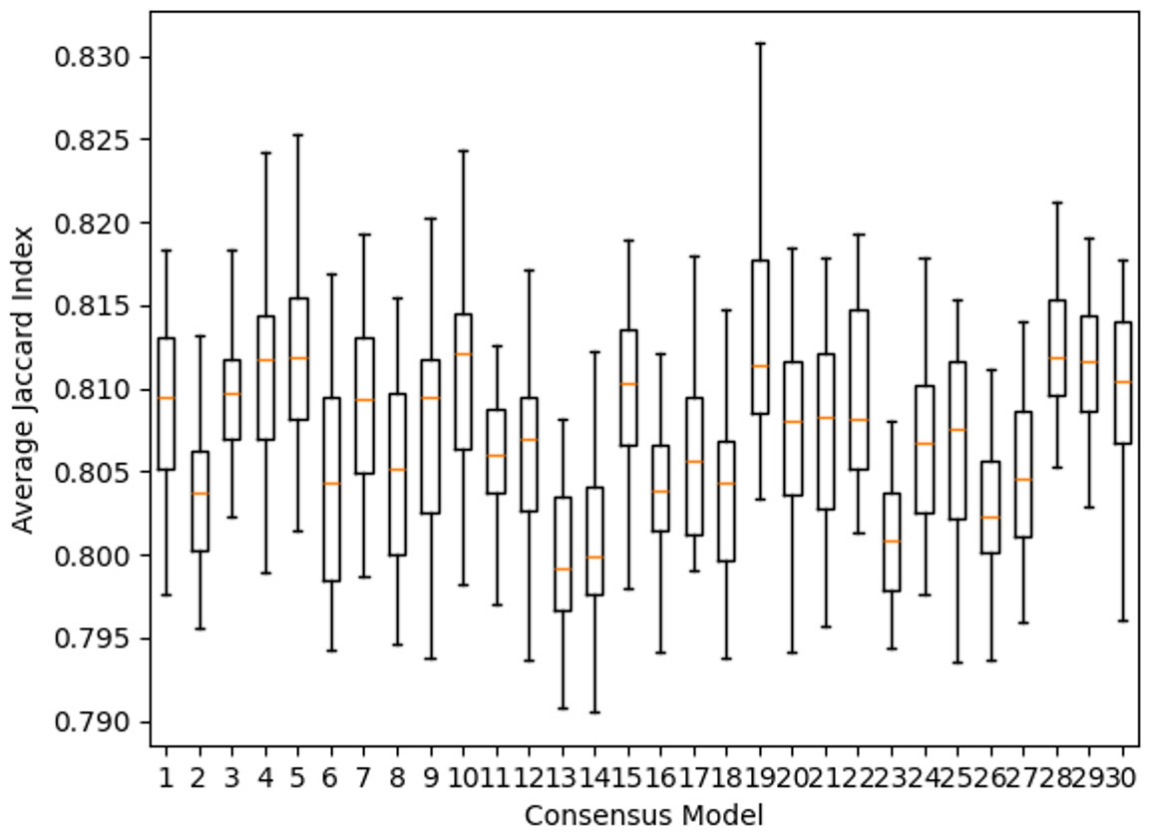
Agreement, based on Jaccard similarity, in rules across different consensus models.

Our analysis of the 30 consensus models showed that they all achieved an average Jaccard index of around 0.8, as shown in Figure 2. There is a fairly high agreement in the left-hand sides of the rules between the models. However, examining the right-hand sides for weight agreement showed a mixed picture depending on how many features are present on the left-hand side.

**Figure 3.**
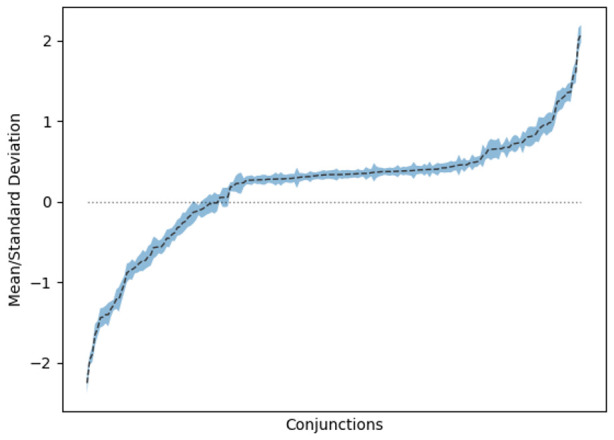
Means and standard deviations for single-premise rules amongst consensus models.

**Figure 4.**
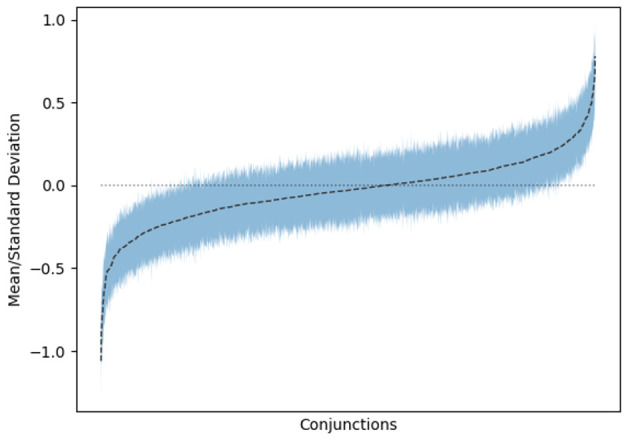
Means and standard deviations for multi-premise rules in amongst consensus models.

Figure 3 shows the means and standard deviations for the weights of rules where there is only one feature on the left-hand side. As can be seen, there is a strong tendency for many of these rules to have either a positive or negative influence on smoking cessation. However, as can be seen in Figure 4, this trend does not continue for the multi-feature rules. We interpret this as a sign that there is not enough data to clearly derive the influence of certain feature combinations.

### Consensus model and prediction interface

**Figure 5.**
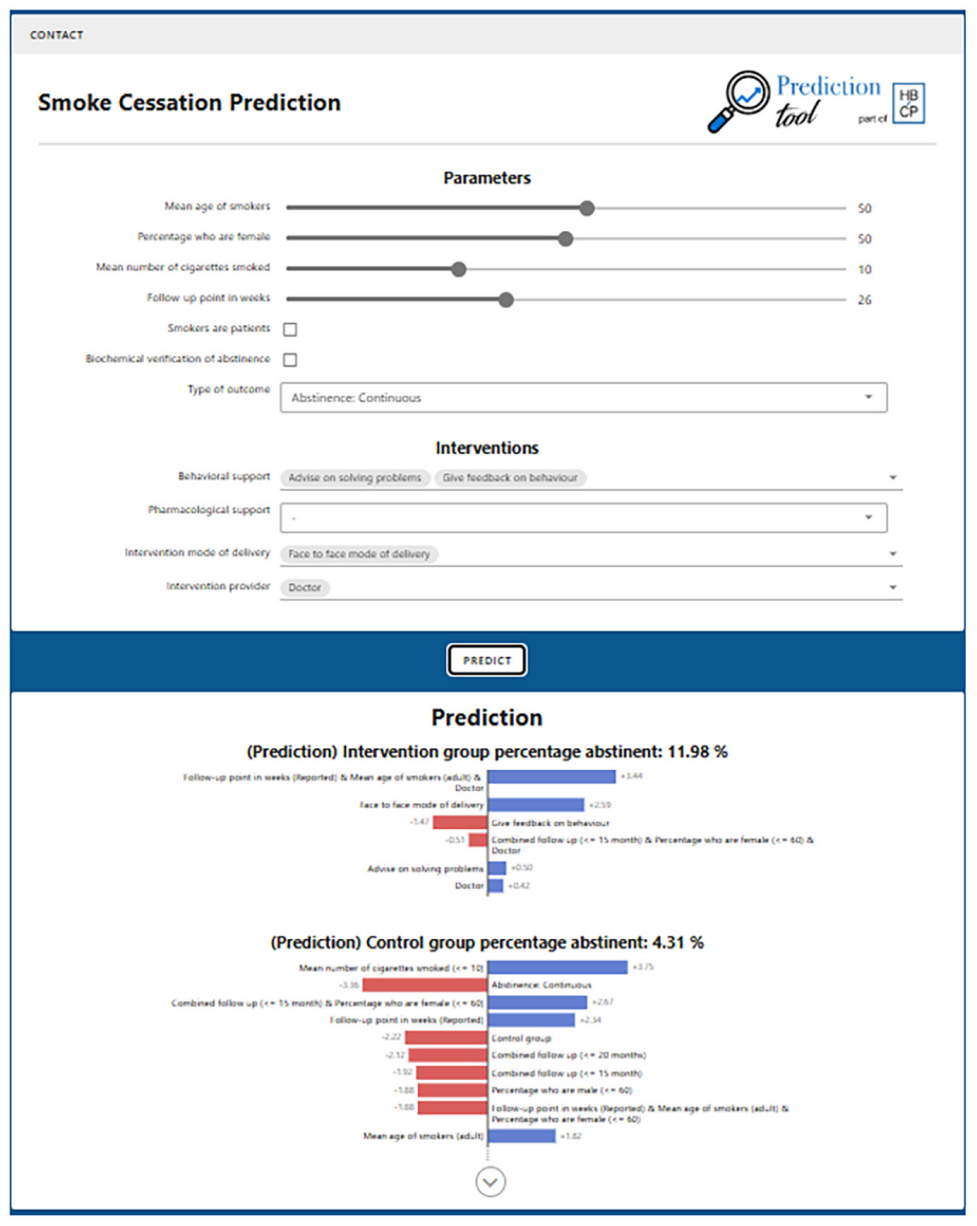
Interactive interface for performing prediction of outcomes of smoking cessation interventions ( https://pred.hbcptools.org/interface/).

The final rule set included 192 rules, including a baseline rule capturing the impact of each feature as well as interaction rules that captured non-linear interactions between features. The prediction interface is deployed here. Figure 5 shows the interface with default values set.

The sliders at the top allow users to set the features of the target population, follow-up point and study methods. Dropdown menus can be used to add features of the intervention, including the BCTs, the intervention packages, brief advice and motivational interviewing, modes of delivery and the intervention source, as well as pharmacological support (NRT or various forms, bupropion, varenicline or placebo). The panel under the sliders shows the rules that are active in the current model for a control group and an intervention group. In the default set-up there are no rules in the intervention group because no intervention features have been added. The rules for the control group reflect the settings for the population, follow-up point and methodology. The coloured bars show graphically whether rules increase or decrease the predicted success rate: blue bars represent an increase and red bars represent a decrease.

The interface shows that in the absence of any intervention features being added, the predicted success rate for the control group of studies was 4.31% and for the intervention group was 7.02%.

The changes in predictions made by the model with varying scenarios accorded with what was expected from prior knowledge. Thus, changing the population age in the default model to 20 years reduced the predicted abstinence rate in the control condition to 2.06% while increasing it to 70 years increased it to 8.27%. Increasing cigarette consumption in the default model to 20 cigarettes per day reduced the predicted abstinence rate from 4.31% to 2.76%. Specifying biochemical verification reduced the predicted abstinence rate from 4.31% to 1.28%. Changing the model from continuous abstinence to point prevalence increased the control group abstinence rate to 6.75%.

Adding the BCTs problem solving, reduce negative emotions, social support (unspecified), reduce prompts and cues and behaviour substitution delivered face-to-face by a health professional increased the predicted intervention abstinence rate from 7.02% to 14.85%. Adding NRT raised this to 20.35%, adding bupropion raised it to 19.67% and adding varenicline raised it to 17.81%.

## Discussion

An ontologically-informed interpretable machine learning model, trained on 82 features of reports of 971 study arms from 405 randomised trials was able to predict smoking abstinence outcomes in novel scenarios with a mean absolute error of 9.15% in cross validation and made predictions of changes in abstinence rates with varying input features that corresponded with expectations from prior knowledge.

Predicting outcomes of behaviour change interventions is extremely challenging but the results provide proof of principle that it can be achieved using transparent machine learning models with a level of accuracy that exceeds that of regression approaches and powerful but uninterpretable deep learning approaches.

The web interface provides a potentially useful tool for professionals, service providers and policy-makers to explore options for the content and delivery of smoking cessation interventions, adding and removing behaviour change techniques and exploring different modes of delivery to establish estimates of likely success rates of different combinations. We have prepared a short video explaining the tool and giving an example of its use ( https://youtu.be/fUvfXmeZhnM?si=tpPdLYVQDZn0nntc). An important future development of the approach would be to create a ‘recommender’ system for a given scenario in which the algorithm would run through a large number of potential combinations for a given smoking population and setting or other constraints (e.g., requiring it to be a web-based intervention) and providing estimated outcomes for, say, the top, 5 combinations).

This study opens the way to a new approach to evidence synthesis in behavioural science in which large numbers of features of studies are used to predict intervention outcomes in untested scenarios. This can be used by policymakers and practitioners when designing interventions to meet particular requirements. The rules generated by models of this kind can be used to develop and test theories of behaviour change.

The mean absolute error achieved in cross validation was higher than is desirable. It is not clear what the upper limit for accuracy is in this domain but many factors will have contributed to the degree of error, of which a high proportion can potentially be addressed.

### Study annotation issues

1.    Potentially important predictive features in study reports were not annotated. These include population features such as mental health ^31^, and level of cigarette addiction ^26^; setting features such as country ^32^; intervention features such as drug dosing ^30^, duration and schedule of contact ^33^, additional BCTs ^29^; and the methodology such as follow-up rates and how missing values were handled.

2.    Inaccuracies in feature coding. The inter-rater reliability of coders was relatively high but far from perfect. Even highly trained annotators can make mistakes during coding and sometimes misinterpret the text.

### Study report issues

3.    Important predictive features were not included in study reports. It has been established that substantial amounts of information on important features of intervention studies are missing from study reports, particularly details of interventions and their delivery ^34^. This could at least partly explain why even without any intervention features included, intervention groups were predicted to have higher abstinence rates than control groups.

4.    Information in reports was presented in a way that was ambiguous or misleading, resulting in a failure accurately to code the features. This and the preceding issue can be addressed by using reporting tools such as the Paper Authoring Tool (PAT) ^35^, a free online tool that provides detailed intelligent prompts for information to be reported and builds a draft paper and a machine readable JSON file that obviates the requirement for third party annotation.

### Data issues

5.    Although the training set was large from the perspective of systematic reviews, for machine learning of large numbers of parameters it was small, and many of the features were only present in a few tens of cases.

6.    Each study, which may have several thousand participants, was represented by only one data point. Potentially this represents a substantial waste of data. Thus, for example, there were several large studies on the effectiveness of e-cigarettes that clearly demonstrated that they could be effective in aiding smoking cessation ^36^, but were not sufficient using our methodology to allow e-cigarettes to be included as a predictive feature. Similarly, it has been established that a combination of nicotine patch and another form of nicotine replacement therapy is more effective than a single form of nicotine therapy alone ^37^, but treating each trial as a single data point meant that we could not attempt to estimate this effect in our model. Having access to individual-level data would be far preferable and would lead to much greater precision in outcome prediction.

7.    In predicting outcomes rather than effect sizes of interventions versus comparators, we were not able to take advantage of the key strength of randomised trials, which is the ability to estimate effect sizes of interventions by randomising other factors that contribute to outcomes. There are analytical meta-regression methods that can combine within- and between-study comparisons to assess effect sizes but these have not yet been developed to be able to predict outcomes using large numbers of features.

8.    The outcome values in the studies used for training and testing would have been subject to sampling error, particularly for the smaller studies. They therefore do not represent a fixed ‘ground truth’ as is often the case for machine learning applications, but a probability distribution the variance of which sets an upper limit on the accuracy of any prediction algorithm.

9.    Findings from randomised trials may not generalise well to clinical or general population contexts. It will be important to extend the data set to real-world prospective studies, case-control studies and quasi-experimental studies.

This study is a first attempt to use machine learning to predict behavioural outcomes in novel scenarios characterised in terms of a large number of features of the study population, intervention, behaviour, setting and methodology, as opposed to effect sizes for intervention packages. It demonstrates a proof of principle that such prediction is feasible while indicating areas that will enable it to be improved, including radical improvements in reporting of intervention evaluations. Given the mean absolute errors and substantive areas for improving prediction identified above, we did not conduct hyperparameter optimisation.

Smoking cessation was chosen as the first use case because of the large number of high-quality trials and relatively homogeneous reporting of outcome behaviours. An important next step will be to extend this approach to other behavioural outcomes such as physical activity, diet, substance use behaviours, infection control behaviours and behaviours related to environmental sustainability.

In future work, to make the prototype system more directly useful for policy-makers and practitioners, we would like to expand the system to incorporate constraints related to feasibilty and cost-related aspects of interventions. This would enable the system to predict optimal intervention packages for a given population in a data-driven fashion based on the available evidence, by maximising the predicted intervention outcome while respecting the specified constraints. However, constraints and feasibility can be context-specific thus the associated constraints may be complex. We also plan to expand the system to take additional relevant factors for both population and context into consideration, such as for example income level and educational level, both relevant for interpreting global evidence.

## Conclusions

We developed a novel interpretable ML approach to synthesise evidence from published intervention evaluation reports and use it in the prediction of outcomes from behaviour change interventions for smoking cessation. This new approach was able to achieve a lower mean absolute error than other ML approaches. A publicly available user interface has been developed and available at https://pred.hbcptools.org/interface/. This study is proof of principle for a methodology for predicting outcomes in novel intervention scenarios in behavioural science. The next steps are to address the factors that currently limit prediction accuracy and to extend the approach to other behavioural outcomes.

## Data Availability

Github: Glauer M, Hastings J. (2023). Predicting outcomes of smoking cessation interventions in novel scenarios using ontology-informed, interpretable machine learning - Source Code. https://doi.org/10.5281/zenodo.8334619 ^20^ This project contains the following underlying data: Data (Folder containing all input data used for training) Data are available under the terms of the Creative Commons Zero "No rights reserved" data waiver (CC0 1.0 Public domain dedication). Open Science Framework: Human Behaviour-Change Project. https://doi.org/10.17605/OSF.IO/EFP4X ^2^ This project contains the following extended data: Supplementary File 1 ( https://osf.io/wpt28) Supplementary File 2 ( https://osf.io/q6n2z) Data are available under the terms of the Creative Commons Attribution 4.0 International license (CC-BY 4.0).

## Software availability

Source code available from: https://github.com/HumanBehaviourChangeProject/semantic-prediction

Archived source code at time of publication: https://doi.org/10.5281/zenodo.8334619 ^20^.

Licence: CC0
