# Supplementary material for: Predicting outcomes of smoking cessation interventions in novel scenarios using ontology-informed, interpretable machine learning
Source: Wellcome Open Res. 2025 Oct 6;8:503. Originally published 2023 Nov 7. [Version 2] doi: 10.12688/wellcomeopenres.20012.2 (PMC12603517; doi:10.12688/wellcomeopenres.20012.2)
Supplement: Peer review history

# Reviewer report: Reviewer response for version 2

**DOI:** 10.21956/wellcomeopenres.27438.r136371

**Published:** 2025-11-10

**Authors:** Kate Frazer

**Affiliations:** 1 University College Dublin, Dublin, Leinster, Ireland

This paper notes the role and errors within systematic reviews of effectiveness and how estimates of rate ratios are used to determine efficacy. The authors of this paper highlight the real-world challenges that exist and that *policymakers, planners and practitioners require predictions about outcomes in novel scenarios where aspects of the interventions, populations or settings may differ.*

 This paper presents a study that aimed to develop and evaluate an ontology-informed, interpretable machine learning (ML) algorithm to predict smoking cessation outcomes using detailed information about interventions, their contexts and evaluation study methods. 

 Overall, this is an excellent article that demonstrates ontologically informed, interpretable ML developments and opportunities to build on this learning and put it into practice. 

 The ML algorithm in this paper predicted cessation rates more accurately than other computational methods.

Is the work clearly and accurately presented and does it cite the current literature?

Yes

If applicable, is the statistical analysis and its interpretation appropriate?

Yes

Are all the source data underlying the results available to ensure full reproducibility?

Yes

Is the study design appropriate and is the work technically sound?

Yes

Are the conclusions drawn adequately supported by the results?

Yes

Are sufficient details of methods and analysis provided to allow replication by others?

Yes

Reviewer Expertise:

Public health and social epidemiology. Tobacco control.

I confirm that I have read this submission and believe that I have an appropriate level of expertise to confirm that it is of an acceptable scientific standard.

# Reviewer report: Reviewer response for version 1

**DOI:** 10.21956/wellcomeopenres.22158.r82739

**Published:** 2024-06-14

**Authors:** John Curtin

**Affiliations:** 1 University of Wisconsin-Madison, Madison, Wisconsin, USA

The manuscript describes a study that aims to develop a machine learning model to predict smoking cessation outcomes associated with    published randomized controlled trials. The authors offer this approach as an alternative to meta-analytic approaches and I agree with this. It is an innovative application of machine learning and this approach generally could offer several advantages beyond the use of meta-analytic techniques.  This includes

 1. A clear and rigorous assessment of the predictive performance of the model in new (held out) data.

 2. The ability to model complex interactions among participant characteristics, components of the interventions, characteristics of the outcome measurement and the research setting.

 3.  The ability to set all of these features to specific values and obtain a predicted score for the outcome.

 4. A potentially interpretable model that may be used for explanation in numerous settings.

 These are all very attractive features of the authors' approach.

 There are also several other key strengths and/or contributions from this study. These include the efforts toward transparency such as the sharing of code and data.  The development of a shiny/web interface to implement the consensus model is exciting.  The consideration of several "baseline" models (multi-level model, base-rate model, deep learning model) to compare the rules based model   against is important to benchmark the performance of the rules model.

 Despite the innovative approach and associated strengths, there are some key issues that could be addressed to increase the clarity of  the results and their associated impact.

 First, I do not agree that the "black box" models are "not interpretable". There have been many advances in recent years to better understand how models that use deep learning or other statistical algorithms (like random forest) make their predictions.  For example, SHAP values can be derived for models using any algorithm (including deep learning) to understand both the global (across all observations) and local (for a specific observation) importance of any features.  Of course, these methods only aid interpretation if   the features (or input layer from the deep learning model) are interpreable but that does appear true in this study given that the features represented RCT characteristics.  

 The authors acknowledge that the performance of their rules model was not high.  However, I do not believe sufficient attention was paid to the implications of the magnitude of error for the model.  The error rate (mean absolute error) was approximately 9%.  Yet the effects of many of the features they used as examples were much less than 9%.  Can it be concluded that any of these features have real effects on the outcome if the magnitude of their effects are less than the noise/error?  

 I appreciated the rigor of using cross validation to evaluate the performance of the rules based model in held-out (test data) to assess how well it would generalize to new data.  However, it is not clear that the error rate from this procedure is a valid estimation of the expected error of the "consensus" model that they implemented because it appears that the consensus model features    were selected and the model trained using different methods than those used for feature selection and training during cross-validation.  If true, this should be discussed in more detail to make clear that the cross validation error estimate may be substantially flawed.  Also, the simpler confirmation that features that were expected to affect the outcome do affect it for the consensus model is not a rigorous assessment of its performance.  

 As mentioned above, the use of baseline/benchmark comparison models in this study is attractive. However, it is not clear how the best  configurations were determined for these other models.  Only one configuration/architecture appears to have been considered for the deep learning model.  How was this decided?  Similarly, only a relatively  shallow random forest (tree depth = 3) with only 50 trees    appears to have been considered for that algorithm.  For each of these algorithms, these decisions about tuning relevant hyperparameters should have been made through the cross validation process.  If not, the performance of these models may have been sub- optimal relative to their potential and this would diminish the value of using them as baseline/benchmark comparisons.

 It is also not clear that any formal statistical comparisons were made between performance of the rules based model and the others.  Without such comparisons, the conclusion that the rules based model "out-performed" the other models does not seem warranted.

 The authors suggest that the rules model might be useful to policy makers and/or clinicians but examples of such uses are not provided.  This statement would be more convincing if accompanied by a few clear examples of how this model could be put to use.

Is the work clearly and accurately presented and does it cite the current literature?

Yes

If applicable, is the statistical analysis and its interpretation appropriate?

Partly

Are all the source data underlying the results available to ensure full reproducibility?

Yes

Is the study design appropriate and is the work technically sound?

Yes

Are the conclusions drawn adequately supported by the results?

Partly

Are sufficient details of methods and analysis provided to allow replication by others?

Yes

Reviewer Expertise:

The use of machine learning and personal sensing methods for precision mental health (personalized interventions) with psychiatric disorders including substance use disorders

I confirm that I have read this submission and believe that I have an appropriate level of expertise to confirm that it is of an acceptable scientific standard, however I have significant reservations, as outlined above.

# Reviewer report: Reviewer response for version 1

**DOI:** 10.21956/wellcomeopenres.22158.r71721

**Published:** 2024-02-13

**Authors:** Claudia F. Nisa

**Affiliations:** 1 Global Health Research Center, Duke Kunshan University, Kunshan, Jiangsu, China

This is a fantastic tool and the work of the UCL team and collaborators is moving forward confidently and rigorously. I accepted to review because I appreciate the high-level behavioral science done by this team, but I am not familiar at all with machine learning. Thus, other reviewers should comment on the technicalities of the procedure.

 I can speak to the conceptual characteristics/ implications of this work. I think this is gold you have in your hands but there is little to no discussion about the good this tool can bring to both the scientific community and policy-makers/ practitioners. Perhaps you are thinking about doing this in a different paper. If possible, a bit more discussion about these points:

 1. This tool opens a whole new world to the empirical test and discussion about complements versus substitute stimuli/  components in interventions. I played with the toll quite a lot and it is possible to see effectiveness go up and down, depending on what you add or take out.

 2. I kept waiting for the gold star combination to be declared by the authors; I believe it may at least be possible by different age groups, or/ and different proportion of females (keeping other rules constant). This would be very informative for policy and practice.

 3. Not sure if that is on your radar, but I would suggest future research to include if possible income level/ educational level, which may bring this tool closer to be relevant in developing countries? Not sure how much data there is from RCTs in developing countries.

 All in all, congratulations.

Is the work clearly and accurately presented and does it cite the current literature?

Yes

If applicable, is the statistical analysis and its interpretation appropriate?

I cannot comment. A qualified statistician is required.

Are all the source data underlying the results available to ensure full reproducibility?

Yes

Is the study design appropriate and is the work technically sound?

Yes

Are the conclusions drawn adequately supported by the results?

Yes

Are sufficient details of methods and analysis provided to allow replication by others?

Yes

Reviewer Expertise:

Behavioral Science

I confirm that I have read this submission and believe that I have an appropriate level of expertise to confirm that it is of an acceptable scientific standard.
